# The rocky road to organics needs drying

**DOI:** 10.1038/s41467-023-36038-6

**Published:** 2023-01-21

**Authors:** Muriel Andreani, Gilles Montagnac, Clémentine Fellah, Jihua Hao, Flore Vandier, Isabelle Daniel, Céline Pisapia, Jules Galipaud, Marvin D. Lilley, Gretchen L. Früh Green, Stéphane Borensztajn, Bénédicte Ménez

**Affiliations:** 1 Université de Lyon, Univ Lyon 1, CNRS UMR5276, ENS de Lyon, LGL-TPE, Villeurbanne Cedex, France; 2 Institut Universitaire de France, Paris, France; 3 Deep Space Exploration Laboratory/CAS Key Laboratory of Crust-Mantle Materials and Environments, University of Science and Technology of China, Hefei, China; 4 CAS Center for Excellence in Comparative Planetology, University of Science and Technology of China, Hefei, Anhui China; 5 Blue Marble Space Institute of Science, Seattle, WA USA; 6 Université Paris Cité, Institut de physique du globe de Paris, CNRS UMR 7154, Paris, France; 7 Université de Lyon, Ecole Centrale de Lyon, LTDS, CNRS UMR 5513, 36, Ecully, France; 8 Université de Lyon INSA-Lyon, MATEIS, CNRS UMR 5510, Villeurbanne, France; 9 School of Oceanography, University of Washington, Seattle, WA USA; 10 Department of Earth Sciences, ETH Zurich, Zurich, Switzerland

**Keywords:** Mineralogy, Organic chemistry, Mineralogy, Geochemistry

## Abstract

How simple abiotic organic compounds evolve toward more complex molecules of potentially prebiotic importance remains a missing key to establish where life possibly emerged. The limited variety of abiotic organics, their low concentrations and the possible pathways identified so far in hydrothermal fluids have long hampered a unifying theory of a hydrothermal origin for the emergence of life on Earth. Here we present an alternative road to abiotic organic synthesis and diversification in hydrothermal environments, which involves magmatic degassing and water-consuming mineral reactions occurring in mineral microcavities. This combination gathers key gases (N_2_, H_2_, CH_4_, CH_3_SH) and various polyaromatic materials associated with nanodiamonds and mineral products of olivine hydration (serpentinization). This endogenous assemblage results from re-speciation and drying of cooling C–O–S–H–N fluids entrapped below 600 °C–2 kbars in rocks forming the present-day oceanic lithosphere. Serpentinization dries out the system toward macromolecular carbon condensation, while olivine pods keep ingredients trapped until they are remobilized for further reactions at shallower levels. Results greatly extend our understanding of the forms of abiotic organic carbon available in hydrothermal environments and open new pathways for organic synthesis encompassing the role of minerals and drying. Such processes are expected in other planetary bodies wherever olivine-rich magmatic systems get cooled down and hydrated.

## Abstract (web summary)

How complex organics form in a prebiotic world remains a missing key to establish where life emerged. The authors present a road to abiotic organic synthesis and diversification in hydrothermal contexts involving magmatism and rock hydration.

## Introduction

In nature, very few organic compounds are recognized as abiotic, i.e., formed by mechanisms that do not involve life^1,2^. Abiotic methane (CH_4_) is the most abundant of those compounds, and can be accompanied by short-chain hydrocarbons (ethane, propane) or organic acids (formate, acetate) in fluids^3–8^ occurring in molecular hydrogen (H_2_)-enriched hydrothermal systems where olivine-bearing rocks are altered via serpentinization reactions^9^ in various geological contexts (i.e., subduction zones, ophiolites, mid-ocean ridges—MOR). Because of this limited variety of small abiotic organic molecules and their strong dilution in hydrothermal fluids, prebiotic reactions cannot easily lead to more complex molecules of biological interest; thus, constituting a limiting factor for a unifying hypothesis for a hydrothermal origin of life on Earth. Without evidence for alternative abiotic organic molecules or pathways and based on an abundance of diverse organic molecules in comets and meteorites^10–12^ (e.g., carbonaceous kerogen-like material, amino acids, polycyclic aromatic, or aliphatic hydrocarbons) many have considered that life’s ingredients had an extraterrestrial origin.

Recent studies of serpentinized rocks along the Mid-Atlantic Ridge (MAR) have highlighted low temperature (T), abiotic formation of aromatic amino acids via Friedel–Crafts reactions catalyzed by an iron-rich saponite clay^13^. Such a process requires a nitrogen source for amine formation and a polyaromatic precursor whose origin remains unknown, and suggests the availability of more diverse abiotic organic reactants than previously expected on Earth, notably in the subseafloor. The discovery of low-T formation of abiotic carbonaceous matter in ancient oceanic lithosphere^14^ also leads to the consideration of new paradigms for organic synthesis pathways within the rocks hosting hydrothermal fluid circulation^15^. Processes leading to such complex, condensed compounds, during rock alteration are unknown but must differ from the mineral-catalyzed Fischer-Tropsch Type (FTT) process that is the most invoked so far in hydrothermal fluids^16–19^ to explain the formation short-chained hydrocarbons. Understanding the variety and formation mechanisms of abiotic organic compounds on Earth, as well as their preservation, has important implications for the global carbon cycle, but also expands the inventory of the forms of carbon available for present-day ecosystems and prebiotic chemistry, and compliments data from extraterrestrial systems^20,21^.

**Fig. 1:**
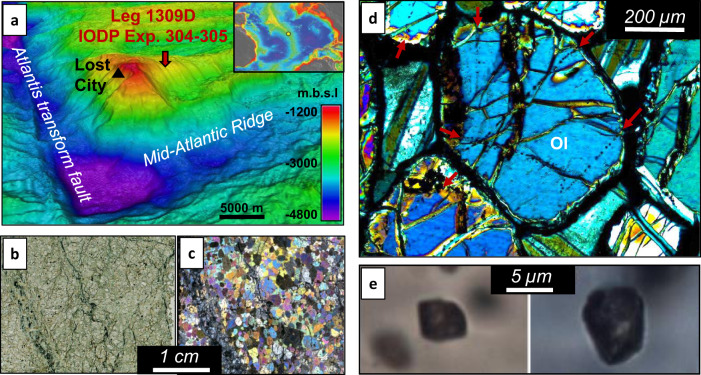
Location and characteristics of the magmatic rock samples. **a** High resolution bathymetric map of the Atlantis Massif hosting the Lost City hydrothermal field. The massif is mainly composed of mantle and mantle-derived magmatic rocks exhumed along the Mid-Atlantic Ridge (MAR) parallel to the Atlantis transform fault (“m.b.s.l.” stands for meters below sea level). The inset shows its location at the MAR scale. **b**, **c** Thin section scans in natural and cross-polarized light, respectively, of a characteristic troctolite sample used in this study and recovered at 1100 meters below sea floor by drilling the Atlantis massif during the IODP Expedition 304–305 Leg 1309D (sample 1309D–228R2). **d** Optical view in transmitted cross-polarized light of olivine (Ol) kernels hosted in the same troctolite. Red arrows show planes of secondary fluid inclusions within olivine crystals. **e** Optical photomicrographs in transmitted plane-polarized light of olivine-hosted multiphasic fluid inclusions.

Here we demonstrate how deep mid-ocean ridge processes can provide an unexpected diversity of abiotic organics as gaseous and condensed phases thanks to water-consuming mineral reactions. Our study focuses on the investigation of olivine mineral microcavities (secondary fluid inclusions (FI)) aligned along ancient fracture planes where circulating fluids were trapped within one of the deepest igneous-rock sections drilled along the MAR, i.e., IODP Hole 1309D, 1400 meter-depth below seafloor – m.b.s.f., at the Atlantis Massif (30°N MAR, IODP Expeditions 304–305, Fig. 1). Five km to the south of Site 1309, Atlantis Massif hosts the Lost City hydrothermal field^22^ where the discharge of abiotic H_2_, CH_4_ and formate have been observed in fluids^19,23^. Within the shallow rock substrate of Hole 1309D (~170 m.b.s.f), abiotic amino acids were identified^13^. At deeper levels (1100–1200 m.b.s.f), olivine-rich igneous rocks such as troctolites are particularly fresh and rich in FIs where they form linear trails of various orientations within olivine grains (Fig. 1b–e). Such FIs are inherited from the first stages of rock cracking and healing during cooling of the lithosphere, allowing the trapping of circulating fluids. Crack-healing of olivine is expected between 600 and 800 °C^24,25^ and at Hole 1309D fluid trapping occurs down to ~700 °C–6000 m.b.s.f. (P~2 kbar)^26^. During cooling, rocks were progressively exhumed below an extensive fault zone up to their present-day position (*P* < 0.3 kbar and T~100 °C^27^).

## Results and discussion

### Diverse organic compounds and nanodiamonds in microcavities

**Fig. 2:**
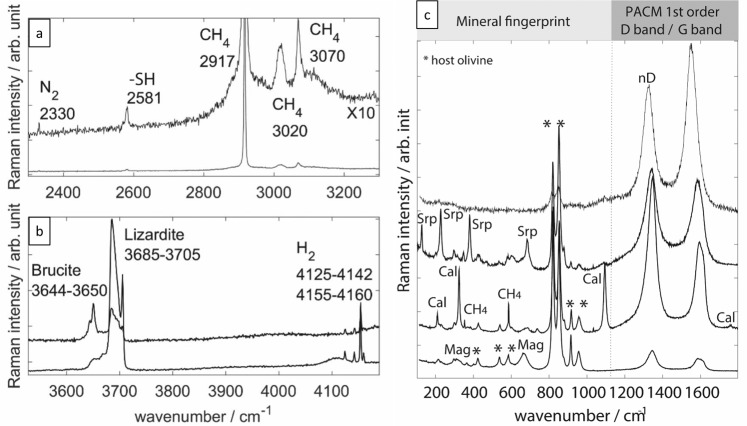
Representative punctual Raman analyses of individual fluid inclusions. They show a large diversity of gaseous^109–111^ (**a**, **b**) and secondary mineral phases^112,113^ and of polyaromatic carbonaceous material (PACM) **(b**, **c**)^33–36^. CH_4(g)_ is well identified by its triplets at 2917, 3020, 3070 cm^−1^ whereas H_2(g)_ raman shifts are found between 4152–4142 and 4155–4160 cm^−1^. N_2(g)_ displays a thin band at 2330 cm^−1^ while the thiol group (-SH) of methanethiol CH_3_SH_(g)_ is observed at 2581 cm^−1^. The PACMs are characterized in their first order region by two broad bands assigned to the disorder (D) band and the graphite (G) band. Few tens of nm-sized nano-diamonds (nD) are identified by the characteristic downward shift of the D band at ~1325 cm^−1^ (ranging between 1313-1332 cm^−1^), its broadening (FWHM-D of 54–70 cm^−1^) and an associated G band near 1550 cm^−1^^41–44^. Interpretation of parameter variability in nD is complex and beyond the scope of the present contribution. Srp serpentine, Cal calcite, Mag magnetite.

Punctual Raman analyses (see Methods) were made on 36 closed FIs in olivine grains forming three troctolites cored at IODP Hole 1309D (intervals 228R2, 235R1, and 247R3). The samples were very fresh and only displayed a localized alteration along thin serpentinized veinlets, underlined by magnetite grains (Fig. 1b, c). All FIs contained H_2(g)_ and/or CH_4(g)_ as well as secondary minerals serpentine (lizardite ± antigorite), brucite, magnetite, or carbonates (calcite or magnesite), as previously observed in similar or ancient rocks^18,28–32^ (Fig. 2). In addition, we documented for the first time in present-day oceanic lithosphere the presence of N_2(g)_, methanethiol (CH_3_SH_(g)_), and variably disordered polyaromatic carbonaceous materials (PACMs) closely associated with secondary minerals in FIs (Fig. 2a, c).

**Fig. 3:**
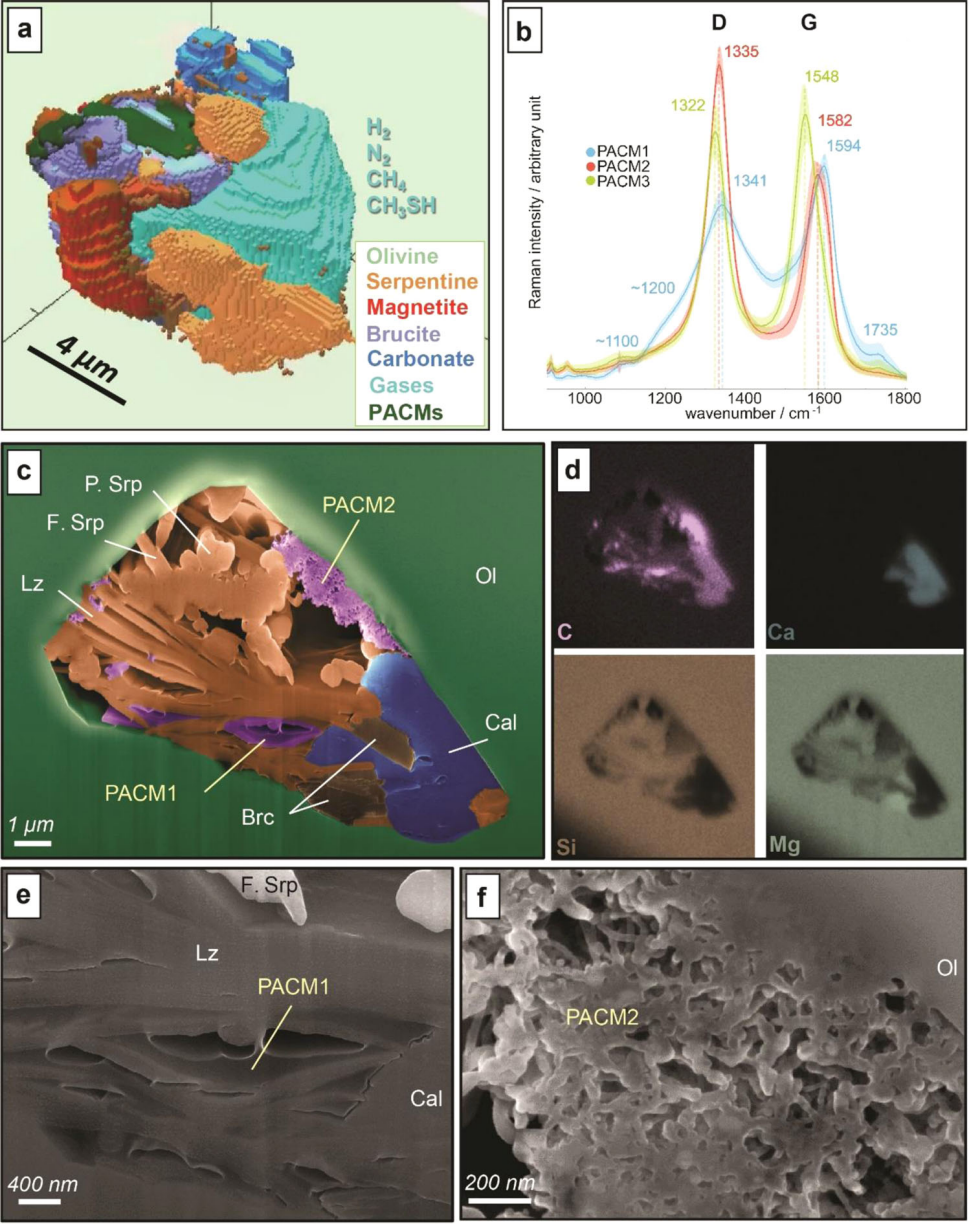
Diversity of gaseous and condensed abiotic organic compounds associated with secondary minerals in single fluid inclusions trapped in olivine minerals of the deep oceanic lithosphere. **a** Three dimensional Raman imaging of fluid inclusion FI3 showing polyaromatic carbonaceous materials (PACMs)^33–36^ coexisting with reduced gaseous species identified as H_2_, N_2_, CH_4_, and CH_3_SH and micrometric serpentinization-derived mineral phases^109,111^ (i.e., serpentine, brucite, magnetite, and carbonate). See also Supplementary Movie 1. **b** Raman spectra highlighting the 3 end-member types of PACMs in the two individual fluid inclusions (FI3 and FI5), all characterized by two broad bands assigned to the disorder (D) band and the graphite (G) band but showing variable position, intensity and width. For each end-member, a mean Raman spectrum is presented (bold line) with the standard deviation (colored shadows). **c** False color scanning electron microscopy (SEM) image of FI5 fluid inclusion freshly opened by focused ion beam milling showing distinct types of PACMs which contrast by their apparent textures: gel-like or mesoporous with nanofilaments are characteristic of PACM1 and PACM2, respectively. **d** Associated elemental mapping using energy dispersive X-ray spectrometry of the olivine (Ol) hosted fluid inclusion allows the identification of PACMs, fibrous polygonal serpentine (F. Srp), lamellar serpentine (lizardite, Lz), polyhedral serpentine (P. Srp), calcite (Cal), and brucite (Brc), as also supported by Raman microspectroscopy (Fig. 2). **e**, **f** Magnified SEM views of **c**, highlighting PACM1 and PACM2, respectively.

To further investigate the nature of the PACMs, high-resolution 3D Raman mapping was carried out on two FIs from one olivine grain of sample 1309D-228R2 (Fig. 1d, e) and were named FI3 and FI5 (Fig. 3a and Supplementary Movie 1). The FI that was richest in PACM (FI5) was then milled and imaged using focused ion beam (FIB)-scanning electron microscopy (SEM) associated with electron dispersive X-ray spectrometry (EDS) (Fig. 3c–f) before being extracted as an ultrathin section (Supplementary Fig. 1) for high resolution transmission electron microscopy (HR-TEM) and X-ray photoelectron spectroscopy (XPS). See Methods for details.

**Fig. 4:**
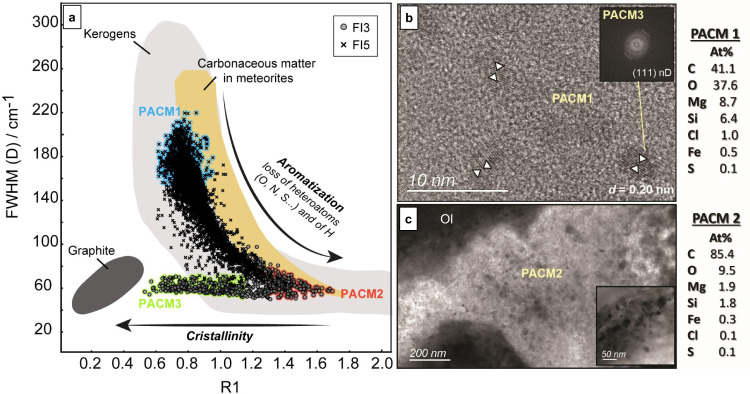
Diversity of the polyaromatic carbonaceous material that displays strong structural and chemical heterogeneities while coexisting at micrometric scale in the two individual fluid inclusions (FI3 and FI5). **a** PACMs heterogeneity as shown by fitting parameters derived from 3D hyperspectral Raman mapping of FI3 and FI5 (e.g. Fig. 3a), namely full width at half maximum (FWHM) of the D (i.e., disorder) band and the relative intensities R_1_ of the D and G (i.e., graphite) bands (=*I*_D_/*I*_G_). The colored data correspond to the data points used to calculate mean Raman signals shown in Fig. 3b. Also reported are the values obtained for kerogens, carbonaceous material in meteorites, and graphite compiled from the literature^33–35^. **b**, **c** High resolution TEM imaging of the PACMs with the qualitative chemical composition of PACM1 and PACM2 measured with energy dispersive X-ray spectrometry. The amorphous, most disordered material (PACM1) plots at the top of the data points in **a** and contains the highest amount of heteroatoms, notably O. The most aromatic material (PACM2) plots at the lower-right end of the graph and is richer in C, tending toward amorphous carbon. The nano-crystalline phase (~5 nm-sized) embedded in PACM1 **b**, and possibly in PACM2 (dotted texture in **c**), has been identified as nano-diamond (nD) both by high-resolution TEM (Fast Fourier Transform of the TEM image in insert) and with Raman (PACM3; Figs. 2a and 3b). PACM3 plots toward the lower-left end of the diagram in graph **a**, where well organized aromatic C skeleton is expected, but graphite is metastably replaced by nD here. *PACM* polyaromatic carbonaceous material, *Ol* olivine, *d* interfoliar distance of the (111) planes in cubic nD.

Quantitative parameters were extracted from 3D hyperspectral Raman data collected on FI3 and FI5 (see Methods) and compared to those of graphite, terrestrial biologically-derived kerogens, as well as carbonaceous matter in meteorites (Fig. 4a)^33–36^. Previous investigations established that the trend followed by terrestrial kerogens and meteoritic carbonaceous matter in such diagrams reflects an increase in thermal maturation during prograde metamorphism. Thermal maturation globally involves organic matter carbonization characterized by a decrease of the full-width at half maximum of the disorder (D) band (FWHM (D))^33,36^. It is materialized by a decrease in heteroatom-bearing groups (e.g., O, N, or S) and aliphatic units, and an increase in the degree of aromaticity. It can be followed by graphitization during which the structural order of the graphitic material increases. This corresponds to a decrease of defects in aromatic planes, characterized by the decrease of the relative intensities R_1_ of the D and graphite (G) bands (R_1_ = *I*_D_/*I*_G_). While such a metamorphic history does not apply in the present context of cooling and exhumation of deep-seated rocks at the Atlantis Massif, this trend is used here to chemically and structurally describe the observed material based on its comparable spectroscopic characteristics. The PACMs contained in our two 3D-imaged FIs cover an unusually large range depicted in Fig. 4a, attesting to an unexpected diversity in chemistry, aromatization degree and structural order at the micrometric scale. PACMs in FI5 alone displays a trend similar to those described in all meteorites, i.e., forming a continuum between 2 end-members, referred to here as PACM1 and PACM2 (see also Fig. 3), reflecting various degrees of aromatization. PACMs of FI3 overlap the FI5 trend but show a complementary trend toward a more structured state defined as PACM3 (see also Fig. 3) with increased crystallinity.

PACM1 displays the most complex structure. In addition to the disorder (D) and graphite (G) bands, two additional contributions are detectable (Fig. 3b). The band at ~1735 cm^−1^ is characteristic of the stretching mode of the carbonyl functional group (C = O), and the shoulder around 1100 cm^−1^ fits well with stretching vibrations of C–O/C–O–C in ether or carboxylic ester functional groups^37^. In PACM1, a shoulder is also visible near 1200 cm^−1^. A similar component has been described in natural and synthetic functionalized carbon systems, while lacking in more carbonized or graphitic materials^38^. Its origin is not well understood, but it was previously attributed to vibrations of C–H/C–C_alkyl_ in aromatic rings^39^. 3D data indicate that PACM1 is spatially well distributed in the FI and is primarily associated with phyllosilicates, corresponding to the gel-like phase wetting serpentine and brucite fibers in FI5 (Fig. 3c–e). HR-TEM examination attests to its amorphous structure and enrichment in O (C/O~1) and in other heteroatoms including metals and S, as shown by associated EDS analysis (Fig. 4b). This agrees with the high level of structural disorder and functionalization deduced from Raman spectra (Figs. 3b and 4a).

Raman and SEM imaging shows that PACM2, observed in both FIs, is localized on olivine walls where it forms a mesoporous texture made of nanofilaments (Fig. 3c, d and f) of ~20 nm in diameter and up to hundreds of nm long. This spongy texture was more difficult to mill under FIB resulting in thicker foils which limited the study of its structure using HR-TEM (Fig. 4c). Associated qualitative EDS analysis shows that PACM2 is made of more than 80% carbon (C/O~9) with traces of the same other elements as PACM1, and confirms that PACM2 is more aromatized than PACM1.

Well-structured nanometric phases, ~5 nm in diameter, are locally observed within amorphous PACM1 (Fig. 4b). These nanoparticles display a lattice parameter *d*~0.20 nm that corresponds to the *d*_111_ of cubic nano-diamond (nD). Raman signals of nD strongly depends on their structure, purity, crystal size and surface chemistry^40,41^, but the smallest ones (<few tens of nm) commonly display a downshift and broadening of the D band due to phonon confinement effects^42–44^ and an additional G band attesting to residual defects and graphitic domains within a surrounding carbon shell^41,43^. PACM3, which plots in the lower-left end of Fig. 4a where most crystalline materials are expected, displays a Raman pattern (Figs. 2c and 3b) similar to nD with a characteristic D band shifted at ~1325 cm^−1^, a FWHM-D of 54–70 cm^−1^ and a G band near 1550 cm^−1^. 3D Raman data of nD (PACM3) are also co-localized with PACM2 that shows a dense and spotted texture made of particles 5–50 nm in diameter, hence attributed to nD (Fig. 4c).

XPS C 1*s* core level spectra was acquired on the whole FIB section of FI5 (Supplementary Figs. 1, 2, and Methods) that contains both PACM1 and PACM2 (Figs. 3c and 4a), spatially unresolved with this method. XPS data reveal a dominant contribution to the PACMs’ structure of C–C/C = C and C–H bonds (~80%), in addition to C–O/C–O–C (~12%) and C = O/O–C = O (~5%) bonds (Supplementary Table 1, ref. ^45^). This confirms previous observations of the dominance of a macromolecular structure with H- and O-bearing functional groups. The remaining contributions correspond to carbon in the form of carbonate (CaCO_3_ here, Fig. 3) and carbide (Supplementary Table 1). The survey spectrum shows the presence of silicon and titanium in small quantities that could form such a carbide (refs. ^46,47^). The latter was not clearly located but it most probably contributes to the nano-particles observed in the C-rich PACM2 (Fig. 4c), together with nD.

Carbon and hydrogen isotopic composition of the CH_4_ contained in fluid inclusions of sample 1309D–228R2 was determined by crushing experiments (see Methods). A minimum concentration of 143 µmol of CH_4_ per kg rock was measured on this sample. ${{{\mathrm{\delta}}}}^{13}{{{\mathrm{C}}}}_{{{{{\mathrm{CH}}}}_{4}}}$ values of −8.9 ± 0.1‰ and ${{{\mathrm{\delta}}}}{{{\mathrm{D}}}}_{{{{{\mathrm{CH}}}}_{4}}}$ of −161.4 ± 1‰ were obtained. They fall within the abiotic range of natural CH_4_^3,31^ and are close to the compositions of CH_4_ venting in Lost City hydrothermal chimneys nearby on the same massif^19^.

### The ideal combination for abiotic synthesis of diverse organics

The nature of the original fluid can be inferred from the current phases found in the FI that attest to in situ reactions with olivine walls. The occurrence of hydrated secondary minerals (serpentine, brucite; Figs. 2 and 3) and of C-, N- and S-bearing phases (N_2,_ CH_4_, CH_3_SH, PACMs, carbonates, carbide, and nD) requires an aqueous fluid enriched in C, N and S, to be trapped as olivine-hosted inclusions. At MOR, such a fluid can be magmatic or seawater-derived, or a mix of both. The fresh character and the Sr isotopic compositions of deep magmatic rocks from the same hole attest of their very limited interaction with seawater^48^ that resulted in late serpentine veinlets, formed after the FIs (Fig. 1b–d). If any, seawater would not be a significant source of carbon to such deep fluid inclusions since dissolved inorganic carbon (DIC) is efficiently removed from seawater at shallower levels by carbonate precipitation, and dissolved organic carbon (DOC) should be rapidly captured in shallow rocks or decomposed in high temperature fluids (T > 200 °C)^49,50^. If few DIC may persist and contribute to the carbon in the fluid inclusion, it is unlikely that any relict DOC, notably the macromolecular component, would remain at the T (600–800 °C) and depth conditions of fluid trapping^24–26^ since seawater-derived fluids would have also undergone boiling and phase separation^51^. Hence, a dominant magmatic origin, resulting from magma degassing, is favored for the fluid trapped in our inclusions as previously proposed for olivine under similar deep crustal conditions^52,53^. Such fluids, exsolved from melts, are dominated by CO_2_-rich vapors that can evolve to more H_2_O-enriched compositions with progressive fractionation^52^. Indeed, at MOR, the source of magma is located in the shallow upper mantle where equilibrium thermodynamic speciation for fluids in the C–O–H–N system strongly favors N_2_ and CO_2_ relative to NH_3_ and to other carbon species considered (CO and CH_4_), respectively^54^. This supports the abiotic, dominantly mantle-derived, origin of the N_2_ and the carbon involved in the various organic compounds observed in the FIs. The absence of CO_2_ and the occurrence of H_2_ and CH_4_ suggests a complete reduction of initial CO_2_ to CH_4_ during fluid-olivine reactions inside the inclusions, at a temperature corresponding to H_2_ production by serpentinization (<350–400 °C), rather than a CO_2_–CH_4_ equilibration at higher temperature. This is consistent with the clumped isotopologue data on CH_4_ from seafloor hydrothermal sites, including Lost City, which imply a formation of CH_4_ at ~250–350 °C^55^. The complete reduction of CO_2_ to CH_4_ also fits the ${{{\mathrm{\delta}}}}^{13}{{{\mathrm{C}}}}_{{{{{\mathrm{CH}}}}_{4}}}$ value thus inherited from the original δ^13^C of magmatic CO_2_.

The main S-bearing species should be SO_2_ with some H_2_S depending on the degassing temperature and H_2_ content of the fluid^56,57^ according to the following equilibrium:

1 $${{{{{{\rm{SO}}}}}}}_{2}+{{{{{{\rm{3H}}}}}}}_{2}\leftrightarrows {{{{{{\rm{2H}}}}}}}_{2}{{{{{\rm{O}}}}}}+{{{{{{\rm{H}}}}}}}_{2}{{{{{\rm{S}}}}}}$$

Accordingly, we argue that the aqueous fluid initially trapped in the FIs was dominantly composed of N_2_, CO_2_, and SO_2_, with minor amounts of H_2_, CH_4_, and H_2_S. Proportions of those species cannot be quantified but a compilation of volcanic gas analyses indicates that the redox state of similar fluids, as defined by O_2_ fugacity (*f*O_2,g_), is usually between the log *f*O_2,g_ set by the fayalite-magnetite-quartz (FMQ) mineral buffer FMQ-1 (1 log unit below FMQ) and the nickel-nickel oxide (NiNiO) mineral buffer NiNiO+2 (2 log unit higher than NiNiO), and their pH is acidic with trace amount of HCl (see Methods).

**Fig. 5:**
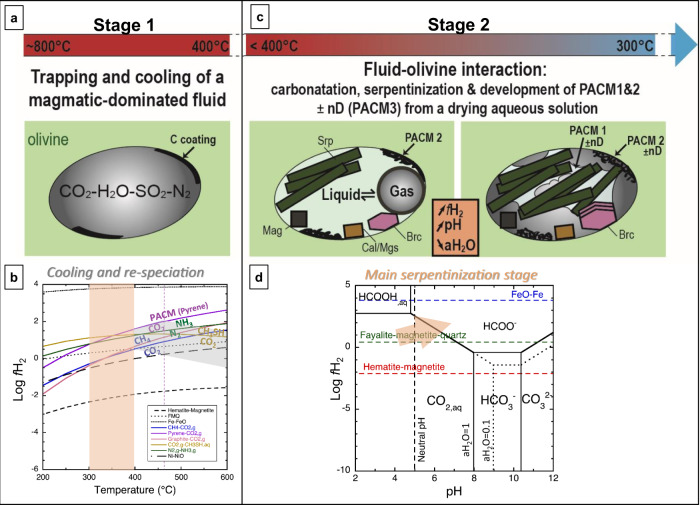
Proposed scenario to account for the chemical and structural diversity of the different types of carbonaceous material in microreactor-like fluid inclusions hosted in serpentinizing olivine from the deep oceanic lithosphere. **a** During **Stage 1**, the trapped fluid cools down to 400 °C at 2 kbar and its speciation evolves as depicted by the gray area in diagram **b**, for a plausible range of initial redox conditions (Supplementary Fig. 3). The path followed by the most reduced fluids (Log *f*H_2_ ≥ FMQ) cross-cut the pyrene-CO_2_ curve between 400 °C and 450 °C, allowing the early formation of pyrene, analogous to the most aromatic PACM observed on olivine walls (PACM2). In these fluids, CO_2_ can also partially convert to CH_3_SH and CH_4_, and N_2_ to NH_3_, before reaching 400 °C; i.e., before serpentinization initiates. The vertical orange area depicts the main serpentinization field (stage 2). **c** During **stage 2**, for T < 400 °C (2 kbar), water becomes liquid and olivine highly reactive with an expected major stage of serpentinization at T between 300 and 400 °C that produces serpentine, brucite, magnetite, and H_2_. Serpentinization advancement rapidly shifts *f*H_2_ and pH  of the solution toward the field of organic acids as schematically drawn by the orange arrow in the speciation diagram, **d**, (350 °C**-**2kbar, methane and methanol suppressed, See Methods and Supplementary Fig. 4), with concomitant carbonate precipitation (calcite or magnesite). This hydration reaction dries out the system leading to condensation of the fluid and formation of PACM1 that wets product minerals and displays varied functional groups bearing O, H, ± S heteroatoms, in agreement with Raman, TEM, and XPS data. nD (PACM3) can metastably form from the amorphous PACM1 and PACM2 during this serpentinization stage^114^. Then, the chemical and structural characteristics of PACMs are expected to evolve with time and during cooling, and contribute to the formation of CH_4_ that was kinetically limited so far. *PACM* polyaromatic carbonaceous material, *aH*_*2*_*O* water activity, set to 1 or 0.1.

A two-step abiotic process of fluid cooling and subsequent fluid-mineral reactions (serpentinization) is proposed to account for our observations in FI as described below and in Fig. 5.

Stage 1–Trapping and cooling of a magmatic-dominated fluid, T ~ 400–600 °C.

Modeling the evolution of such a fluid during cooling from 600 °C to 400 °C at 2kbar (Fig. 5b, Supplementary Fig. 3a and Methods) shows that the most reduced fluids (Log *f*H_2_ ≥ FMQ) favor CH_4_, CH_3_SH, and graphite below ~550 °C, and NH_3_ below ~450 °C (Fig. 5, stage 1) if kinetics are favorable. The same fluids also first crosscut the pyrene-CO_2_ equilibrium near 450 °C, showing the possibility to form early aromatic materials such as pyrene, used here as a simple analog for PACMs (Fig. 5, stage 1). Deposition of carbonaceous films on freshly-cracked olivine surfaces by condensation of C–O–H fluids during abrupt cooling to 400–800 °C has been described experimentally^58^, inspired by observations of olivine surfaces in basalts and xenoliths^59,60^. In these experiments, the carbonaceous films consisted of various proportions of C–C, C–H, C–O bounds and carbide depending on the redox conditions and final temperature. In our FIs, deposition on olivine walls of the most aromatic material (PACM2), possibly associated with carbides, can be initiated by a similar surface-controlled process^61^ (Fig. 5, stage 1). The initial chemical and structural characteristics of this material are unknown since they probably changed in the FI during the subsequent evolution of physico-chemical conditions (stage 2).

Stage 2 – Serpentinization and formation of various metastable organic compounds. Once T falls below 400 °C, fluid water becomes both liquid and gaseous and olivine is prone to serpentinization therefore leading to the formation of serpentine, brucite, magnetite and H_2_ (Fig. 5, stage 2)^9^. Serpentinization also increases the pH of the fluid that first equilibrates with CO_2_, allowing carbonate precipitation. Previous modeling of these reactions in similar FIs have considered a seawater-derived aqueous fluid variably enriched in CO_2(aq)_^18^. Since PACM, CH_3_SH or N_2_ were not reported, these species were not included in the previous models, but increasing levels of H_2_ were predicted between 400° and 300 °C, shifting the system by more than 2 log*f*H_2,g_ units to highly reducing conditions, allowing CH_4_ formation via reaction (2). Reaction (2) is thermodynamically favored with decreasing T and water activity (aH_2_O) but its slow kinetic at T < 400 °C^16^ needs to be overcome by long residence times (thousand years) of the fluids in FIs^53^.

2 $${{{{{{\rm{CO}}}}}}}_{2}+{{{{{{\rm{4H}}}}}}}_{2}\leftrightarrows {{{{{{\rm{CH}}}}}}}_{4}+{{{{{{\rm{2H}}}}}}}_{2}{{{{{\rm{O}}}}}}$$

However, olivine serpentinization is very fast at optimum conditions near 300 °C and can be completed in few weeks to months^62^. At this short time scale, the kinetic inhibition of methane formation prevails and metastable organic compounds are predicted to form, including aliphatic and polyaromatic hydrocarbons (PAHs), organic and amino acids or condensed carbon^61,63,64^. Suppressing CH_4_, we have modeled the redox evolution of the fluids in our FIs during serpentinization (Methods and Supplementary Fig. 3). *f*H_2_ also increases of ~2 log units between 400 °C and 300 °C, buffered here by the precipitation of PACM (analog to pyrene). The increase of *f*H_2,g_ and pH due to serpentinization can progressively shift the carbon speciation in solution toward the fields of organic acids (e.g., formic acid, reaction (3) and orange arrow on Fig. 5d, or acetic acid, Supplementary Fig. 4) that are common species in serpentinizing systems^1,6,23^. These fields widen with decreasing water activity (*a*H_2_O) and T (Fig. 5b, Supplementary Fig. 4).

3 $${{{{{{\rm{CO}}}}}}}_{2}+{{{{{{\rm{H}}}}}}}_{2}\leftrightarrows {{{{{{\rm{HCOO}}}}}}}^{-}+{{{{{{\rm{H}}}}}}}^{+}$$

Carbon speciation of the fluid is probably even more complex, notably with contributions of others O-bearing reduced species (e.g., CO, aldehydes or alcohols)^1,65^. Reduction of N_2_ to ammonia is also favored with increasing *f*H_2_,g (e.g. Fig. 5, stage 1), making possible the formation of CN-containing organic species. The abiotic formation of CH_3_SH may be initiated earlier from the fluid initially trapped (Fig. 5, stage 1) but can continue during serpentinization via reaction (4)^66^; organic acids being potential intermediate products^1^.

4 $${{{{{{\rm{CO}}}}}}}_{2}+{{{{{{\rm{H}}}}}}}_{2}{{{{{\rm{S}}}}}}+{{{{{{\rm{3H}}}}}}}_{2}\leftrightarrows {{{{{{\rm{CH}}}}}}}_{3}{{{{{\rm{SH}}}}}}+{{{{{{\rm{2H}}}}}}}_{2}{{{{{\rm{O}}}}}}$$

Occurrence of thioester functions is also possible through condensation of available thiols and carboxylic acids according to reaction (5):

5 $${{{{{\rm{RSH}}}}}}+{{{{{\rm{R}}}}}}{\prime} {{{{{{\rm{CO}}}}}}}_{2}{{{{{\rm{H}}}}}}\to {{{{{\rm{RSC}}}}}}({{{{{\rm{O}}}}}}){{{{{\rm{R}}}}}}{\prime}+{{{{{{\rm{H}}}}}}}_{2}{{{{{\rm{O}}}}}}$$

More generally, hydrothermal conditions favor dehydration reactions of organic compounds such as amide or ester formation from carboxylic acids^67^, in addition to organic functional group transformation reactions^68^, which both considerably enlarge the range of organic compounds that can be formed. The absence of liquid water in the FI today attests to the full consumption of water during serpentinization of the olivine walls that should have progressively enhanced reactions (1) to (4) and condensation reactions (e.g., reaction (5)). Based on the structural and chemical characteristics of PACM1 (Figs. 3 and 4), and its “wetting” texture on hydrous minerals (Fig. 3c, e), we propose that this complex gel-like material was formed by condensation of the fluid enriched in organics during this serpentinization-driven drying stage.

Metastable phases such as PACM1 and PAMC2 are prone to evolve after their formation. Here, they seem to serve as organic precursors for nD nucleation under the low P–T conditions of modern oceanic setting, similarly to higher P–T processes in subduction zones (>3 GPa)^69^. Occurrence of nDs within the stability field of graphite have been previously described in ophiolites under similar conditions^70^ and at higher T (~500–600 °C)^71^, as well as experimentally^72^. It has also been predicted by thermodynamic models^73^. Our results first suggest that nDs formation in such low P–T environments (≤2 kbars–400 °C) possibly occurs via an intermediate, amorphous, organic material. CH_4_ and possibly other hydrocarbons^28^ can also form later in FIs from reaction (2) or from further dehydration^74^ and hydrogenation^75^ reactions of PACMs simplified by pyrene (C_16_H_10_) in the following reaction.

6 $${{{{{{\rm{C}}}}}}}_{16}{{{{{{\rm{H}}}}}}}_{10}+27{{{{{{\rm{H}}}}}}}_{2}\leftrightarrows 16{{{{{{\rm{CH}}}}}}}_{4}$$

### New routes for abiotic organic synthesis in the Earth primitive crust and beyond

Considering the geological context of such systems, our observations indicate that the timely interplay between magmatic degassing and progressive serpentinization is an ideal combination for the abiotic synthesis of varied gaseous and condensed organic compounds. Fluid inclusions have long been recognized as a major source of H_2_ and CH_4_ in fossil and active oceanic lithosphere^18,30,31,53^, but the discovery of the new compounds has further implications. The likelihood that fluid inclusions can be opened during lower-T alteration processes at shallower levels in the oceanic crust, render the components trapped in the inclusions available for further diversification and complexification that can benefit prebiotic reactions. Ingredients, gathered and preserved in olivine pods, can suddenly be released in an environment that is far from the original equilibrium conditions. Such a high degree of disequilibria favors the production of many additional organic compounds and promotes the development of chemotrophic microbial metabolisms^76,77^. As an example, similar FIs could have provided nitrogen and aromatic precursors required for the local synthesis of abiotic amino acids that are described in the shallower part of the same drill hole^13^. PACMs may also be the locus of further precipitation of carbonaceous material assisted by mineral reactions at low-T^14^. The studied FIs also provide the first evidence for an abiotic source of CH_3_SH in present-day oceanic rocks where a thermogenic origin was favored up to now^66^. Availability of CH_3_SH and organosulfur compounds such as thioesters may be crucial to initiate proto-metabolisms in primitive hydrothermal systems^66^. In modern systems, such FIs may also provide nutrients for hydrocarbon degrading micro-organisms that have been revealed by genomic studies in magmatic rocks at various depths in IODP Hole 1309D^78^.

H_2_ and CH_4_ enriched alkaline environments created by low T serpentinization have been recognized as providing some of the most propitious conditions for the emergence of life^79–81^. Our results strengthen this hypothesis by highlighting new reaction routes that encompass the progressive time-line of geologic events in such rock systems. Unexplored prebiotic reaction pathways based on similar processes may have occurred in the primitive Earth and on Mars where hydrothermal environments rooted on olivine-rich magmatic rocks (e.g., komatiites on Earth) are thought to be widespread^82–85^. The more reduced state of the mantle on early planets should have favored reduced species^12,86,87^ in the percolating magmatic fluids. Some studies of Martian meteorites have already suggested synthesis of PACM on Mars in relation to combined magmatic and hydrothermal processes^12^. This may be extrapolated to other planetary bodies such as icy moons where serpentinization has become a focus of attention^88,89^.

## Methods

### Transmitted light microscopy

Optical imaging of rock thin sections (30 µm thick) have been done under plane-polarized and cross-polarized light using a Leica transmitted light microscosope.

### Micro-Raman spectroscopy

We acquired all the individual Raman spectra and 3D hyper-spectral Raman images (3D HSR images) with a LabRam HR evolution from Horiba™ manufacturer and a 532 nm DPSS laser. The laser beam was focused onto the sample with an Olympus ×100 objective. The probe spot has a diameter of around 0.9 μm. We used 600 grooves/mm grating to collect Raman spectra in two wavelength ranges, from 120 to 1800 cm^−1^ and from 2500 to 3800 cm^−1^. The first one is associated to the Raman fingerprint of minerals and covers the first order region of PACM with D and G bands. The second one presents hydroxyl’ stretching bands of phyllosilicates, hydrated oxides, CH_4_ stretching modes and the second order of PACM.

With four acquisitions of 500 ms per spectrum, the recording of a 3D HSR image is up to 39 h per spectral range. That means, twice for a full 3D acquisition of IF3 and almost 32 h for IF5. We minimized this time by scanning laser beam instead of shifting the position of the sample with the holding stage. We retain the true confocal performance of the microscope by using the DuoScan® hardware module in stepping mode. The laser was stepped across the sample in X and Y direction by two piezoelectric mirrors. The surface map has a small and high accuracy step, down to 250 nm in our case. Then stacking 2D HSR images from the surface and down in the hosting olivine with Z steps of 250 nm, we composed a 3D image of the fluid inclusion. After data preprocessing of the Raman spectra (see below explanations), we powered these images and 3D animations with the 3D Surface and Volume Rendering (3D SVR) application for LabSpec6®. Assuming minerals and gases are here transparent and our microscope is confocal, 3D shapes can be rendered by association of a color channel to a Raman signature. We used filters to remove voxels which have low color intensity and thresholds to control the transparency.

The preprocessing data treatment differs between the 3D HSR images and the Raman signature of the PACM. In regards to the images, we operated the following sequence of preprocessing: (1) extraction of the relevant wavenumber range, (2) removal of extremely low and high signal corresponding either to low Raman diffusion or to high luminescence, (3) correction of the background with a polynomial base line.

The first order Raman signature of the PACM was extracted from the whole spectra (more than 50,000 per image) acquired during 3D HSR images recording. We used a homemade algorithm with the Matlab® software to extract this Raman signal from each spectra and perform an iterative data fitting with the PeakFit Matlab® application tool peakfit.m^90^. We applied the same procedure as in Quirico et al. (2014)^91^. The two Raman bands D and G were fitted with the so-called Lorentzian-Breit-Wigner-Fano (LBWF) spectral model^36^. Raman spectral parameters characterizing the PACM were extracted: width at half maximum (FWHM-G, FWHM-D), peak position (wG, wD) and ratio of peak intensity R_1_ (ID/IG) with a GOF (Goodness of fit). This parameter was used to remove fits with low RMS fitting error and R-squared. Eventually, we kept on working with typical batches of 600 up to 10,000 spectra with spectral parameters associated. The following table provides characteristic parameters of the averaged PACM end-members Table 1.

**Table 1:** Raman characteristic parameters of the averaged PACM end-members

Averaged data	wD	wG	R_1_	FWHM D
Group 1	1347	1592	0.77	180
Group 2	1332	1587	1.43	62
Group 3	1323	1555	0.78	61

Data mining and visualization of spectral parameters in a workflow were powered with the software Orange^92^. We concatenated the Raman spectral parameters of the two IF to plot the diagram representing FWHM-D as a function of R1. We selected 3 groups of data as endmembers in this diagram to discuss spectral properties of each one and localization between each other in the inclusion.

### Focused Ion Beam milling (FIB) and Scanning Electron Microscopy (SEM)

After being located by transmitted light microscopy and analyzed by Raman spectroscopy, fluid inclusions within the thin section sample were opened by using a FIB - SEM workstation (NVision 40; Carl Zeiss Microscopy) coupling a SIINT zeta ionic column (Seiko Instruments Inc. NanoTechnology, Japan) with a Zeiss Gemini I electronic column. For FIB operation, the thin section was coated with a carbon layer of about 20 nm by a carbon coater (Leica EM ACE600) to prevent electrostatic charging.

First, a platinium coating was deposited with the in-situ gas injection system to define the interest region and to protect the surface from ion beam damage. Prior to milling and imaging, a coarse trench was milled around the region of interest to a depth of 30 µm. The inclusions were closed and not visible on the surface of the sample. The abrasion was therefore done progressively with FIB parameters adjusted to 30 kV and 10 nA until breaking through and obtaining a cross-section of the inclusion.

Subsequently, the observations were performed using backscattered electrons with the so-called Energy and angle selective BSE detector (EsB) and secondary electrons with the Secondary Electrons Secondary Ions detector (SESI). These experiments were operated at 15 kV and in high vacuum. Chemical composition of solids in fluid inclusions was obtained simultaneously by EDX analyses using an Aztec Oxford system (EDS Oxford Instruments Aztec-DDI detector X MAXN 50).

The studied cross sections were then extracted and thinned to a thickness of 100 nm by the ion beam following the lift-out method.

### Transmission electron microscopy (TEM)

The structural organization of these thin foils was investigated by TEM. A 2100 JEOL operating at 200 kV was used to study precisely the carbon-rich regions within the fluid inclusions. A STEM (scanning transmission electron microscopy) module coupled with a EDX XMAX 80 mm^2^ system (Oxford Instruments) allowed the acquisition of images in annular bright field and a precise chemical analysis of the solid and condensed organic phases in the fluid inclusions.

Fast Fourier Transform analysis of high-resolution images of nano-diamond-rich areas were used to determine cell parameter of the ~5 nm-size particles using Digital Micrograph software©.

### X-ray photoelectron spectroscopy (XPS)

XPS analyses were carried out ate the Ecole Centrale de Lyon (France) on a PHI 5000 Versaprobe II apparatus from ULVAC-PHI Inc. A monochromatized AlKα source (1486.6 eV) was used with a spot size of 10 µm. A charge neutralization system was used to limit charge effect. The remaining charge effect was corrected fixing the C–C bond contribution of C1*s* peak at 284.8 eV. Before acquisition of the spectra, a short Ar ion etching was performed (250 V, 1 min) to limit the presence of adventitious carbon on the surface. C1*s* spectra were obtained using a pass energy of 23.5 eV. All the peaks were fitted with Multipak software using a Shirley background. Quantification was carried out using the transmission function of the apparatus and angular distribution correction for a 45° angle. Sensitivity factors were extracted from Wagner et al., (1981)^93^ in which they integrate cross section and escape depth correction.

### Extraction and isotopic analyses of CH_4(g)_

A portion of the studied rock sample was initially crushed with a stainless steel mortar and pestle and sieved to collect 1–2 mm chips. These chips were then heated at 60 °C under vacuum to remove surficial water. Approximately 0.23 g of these chips were placed into a hydraulic rock crusher with a continuous He stream similar to that of Potter and Longstaffe (2007)^94^ and the crusher activated several times until the CH_4_ signal approached that of the blank. The gas released by crushing was focused on a Porapak Q filled quartz capillary trap held at liquid nitrogen temperature. Gases were released from the trap by moving it out of the liquid nitrogen and into a 150 °C heating block.

The released gases were separated on a HP 6890 gas chromatograph fitted with an Agilent Poraplot Q column (50 m, 0.32 mm wide bore, 10 μm film) temperature programmed from −30 to 80 °C. The column effluent was fed into an oxidation oven containing NiO, CuO and Pt catalysts where the reduced gases were converted to CO_2_. Following the oxidation oven, the gases entered a Thermo Fischer Delta V isotope ratio mass spectrometer (IRMS). Data reduction was performed by comparing an in house CH_4_ isotope standard to Indiana University Biogeochemical Laboratory CH_4_ standards #1, #2, #5, and #7.

### Thermodynamic modeling

Equilibrium reaction constants at elevated temperatures and pressures are used to construct the equilibrium speciation diagram (Fig. 5 and Supplementary Fig. 3 and 4). For the aqueous species, we used the Helgeson–Kirkham–Flowers equations and predictive correlations to calculate the Gibbs free energies of formation at high temperatures and pressures^95–97^. The calculations were conducted with the Deep Earth Water (DEW) Model^98^. The Gibbs free energies of formation of minerals and solid condensed carbons at high temperatures and pressures were calculated using the SUPCRT92b code, an adaption of SUPCRT92^99^.

Thermodynamic data files used in the calculations were built using data for aqueous species from Shock et al. (1997)^96^, and minerals from Berman (1988)^100^, Berman and Aranovich (1996)^101^, and Sverjensky et al. (1991)^102^. We adopted the thermodynamic properties of CH_3_SH_,aq_ from Schutle and Rogers (2004)^103^, which are consistent with Shock et al. (1997)^97^. We also included the thermodynamic data of condensed aromatic organic carbons of Richard and Helgeson (1998)^104^, which are consistent with Berman (1988)^100^.

To simulate fluid-rock reactions, we applied purely chemical irreversible mass transfer models^105^ to simulate reactions between a cooling magmatic-dominated fluid and olivine. We consider the system as progressive alteration of olivine in a closed system in which there was always the reaction affinity for the alteration of olivine by water. We set 30 moles of olivine (Fa15Fo85) reacting with 1 kg water, so the approximate water:rock = 1:4.5. It represents a low W/R ratio relevant with the geological settings observed here (very limited fluid captured as olivine inclusions). The dissolved salts of Ca, Mg, Fe, C, Si, N, S were considered in the calculations as well as all available minerals. All the calculations were carried out with the aqueous speciation, solubility, and chemical mass transfer codes EQ3 & EQ6 which have been recompiled from a traditional version^106^ for the purpose of simulating temperature and pressures higher than water saturation conditions, using thermodynamic data files prepared as described above. The codes are accessible freely to the public through the Deep Earth Water community (http://www.dewcommunity.org/). We first simulated the volcanic gas and starting fluid using EQ3 code. We then let the gas cool down to 400 °C before reacting with olivine in a continuous cooling (<400 °C) and enclosed system (2000 bars), mimicking the high-temperature and low-pressure environment where the fluid inclusions formed. It is within the T range of fluids when they are trapped in the inclusions.

The cooling rate is set by the following equation in the model input:

7 $${{{{{\rm{temp}}}}}}\,{{{{{\rm{C}}}}}}={{{{{\rm{temp}}}}}}\,{{{{{{\rm{C}}}}}}}_{0}+{{{{{\rm{tk}}}}}}1\ast \xi+{{{{{\rm{tk}}}}}}2\ast {\xi }^{2}+{{{{{\rm{tk}}}}}}3\ast {\xi }^{3}(0\le \xi \le 1)$$

where temp C_0_ represents the initial temperature in °C; ξ represents the reaction extent; tk1, tk2, and tk3 are three parameters. Here, we set tk1 = −200 for the two cooling calculations: 600–400 °C (without olivine) and <400 °C (with olivine); we used the first cooled fluid (at 400 °C) as the starting fluid to react with olivine for the second stage cooling calculation.

Volcanic gas is mainly composed of steam H_2_O, CO_2_, and H_2_, with other trace gases^107,108^. The composition of the volcanic gas varies depending on several geological factors, including the extent of degassing of the magma, redox state, and temperature and cooling history^108^. Under the circumstances of this study, the simulation used volcanic CO_2,g_ as the only carbon source. Provided the reported CO_2_/H_2_O ratio in volcanic gases, we set the starting CO_2_/H_2_O ratio as 0.3 in our starting fluid.

Compilation of the volcanic gas indicated that the redox state of volcanic gas is between the log *f*O_2,g_ values set by fayalite-magnetite-quartz (FMQ) mineral buffer minus one log unit (FMQ-1) and nickel-nickel oxide (Ni/NiO) mineral buffer plus two log unit (Ni/NiO+2) (Symonds et al., 1994). In our simulation, we set log *f*O_2,g_ of the starting fluid equal to these two values, representing two boundary cases (Supplementary Fig. 3). As the starting fluid would dissolve high pressures of CO_2,g_ and trace amounts of HCl and S gases^107,108^, the starting pH would be acidic. The neutral pH at 600 °C and 2 kbars is 5.3. Therefore, in our simulation, we set the initial pH as 4 to represent an acidic condition.

## Supplementary information

Supplementary Information

Description of Additional Supplementary Files

Supplementary Movie 1

## Data Availability

The data supporting the findings of this study are available within the paper and its Supplementary Information. Any additional information is available from the corresponding author upon request.
