# Supplementary material for: The rocky road to organics needs drying
Source: Nat Commun. 2023 Jan 21;14:347. doi: 10.1038/s41467-023-36038-6 (PMC9867705; doi:10.1038/s41467-023-36038-6)
Supplement: Supplementary file 1 — Supplementary Information [file 41467_2023_36038_MOESM1_ESM.pdf]

Supplementary Information

Supplementary Table 1. Results of the X-ray photoelectron spectrometry spectrum deconvolution for C 1*s* core level. FWHM, full width at half maximum.

	**Position (eV)**	**Assignment**	**Shift (eV)**	**FWHM (Ev)**	**Contribution to total area (%)**
**C1**	282.1	Carbide [112,113]		1	2.8
**C2**	284.8	C-C, C-H [114]	2.66	1.65	78.9
**C3**	286.4	C-O, C-O-C[114]	4.29	1.65	12.0
**C4**	288.2	C=O, O-C=O [114]	6.07	1.65	4.9
**C5**	290.4	Carbonate [114]	8.27	1.65	1.4

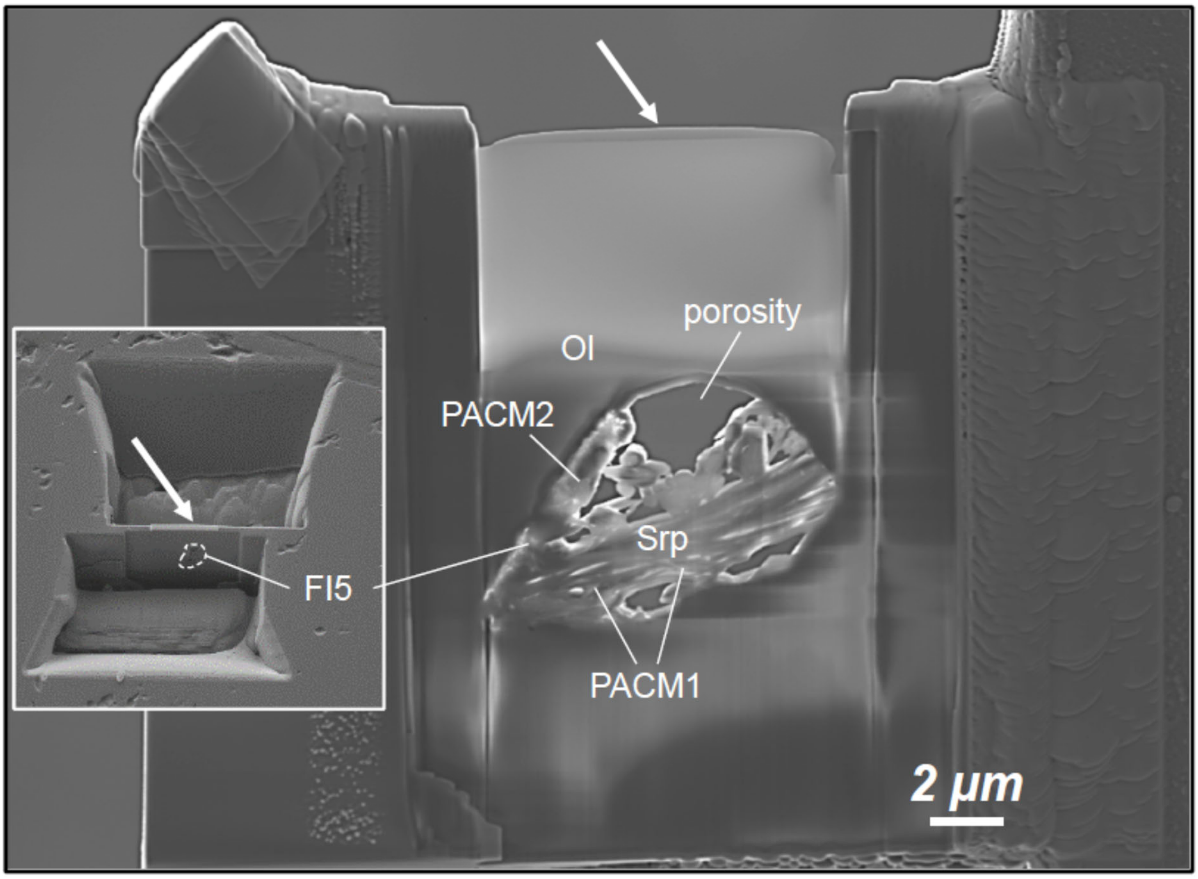

Supplementary Figure 1 | SEM views (secondary electron mode) of the ultrathin foil milled for TEM and energy dispersive X-ray spectrometry investigations of the FI5 fluid inclusion solid content. The left inset shows the step-cut cross-sections on both sides of the foil during excavation before extraction and thinning. The arrow shows the edge of the foil. PACM = polyaromatic carbonaceous material, Srp = serpentine, Ol = olivine.

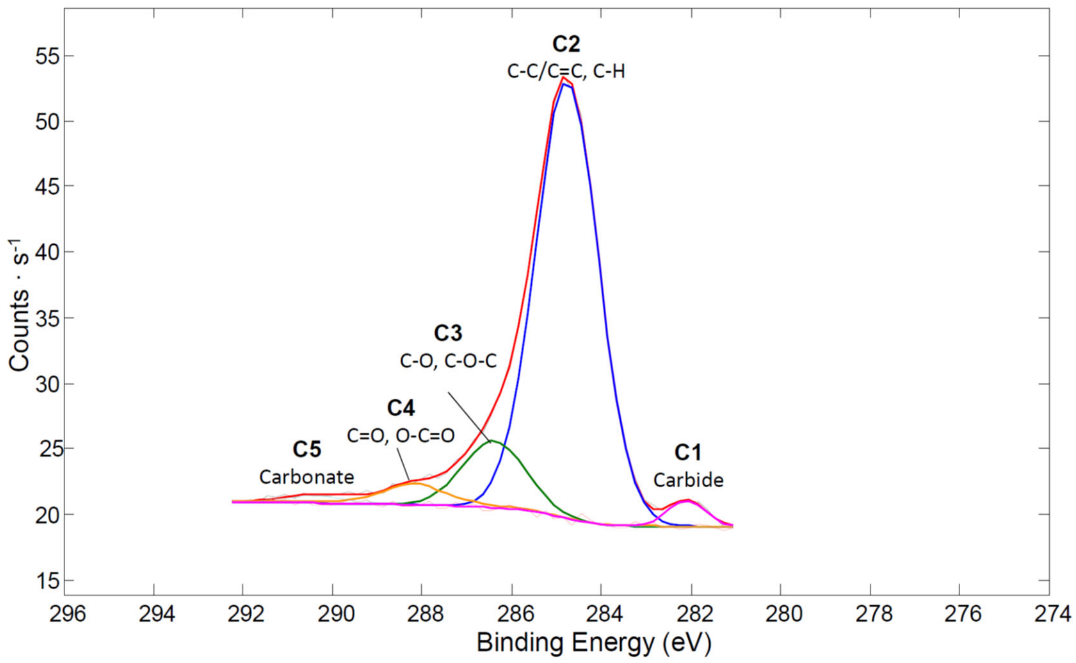

Supplementary Figure 2 | X-ray photoelectron spectrum for C 1*s* core level (black line) collected on the ultrathin foil milled at the location of FI5 (Supplementary Fig. 1) using a 10µm spot size. Deconvolution are reported as colored lines. It shows 5 contributions whose characteristics are reported in Supplementary Table 1. Energy calibration of the spectrum was made using a single C2 contribution at 284.8 eV attributed to aliphatic *sp*3 C-C, C-H bonds. However, the width of the peak is compatible with the presence of aromatic *sp*2 C bonds. Energy resolution and signal-to-noise ratio are too low to properly assert this hypothesis. Organosulfur, organometallic, and N-bearing compounds were not detected by XPS on such a small (~5 µm) and thin (<100 nm) rock section.

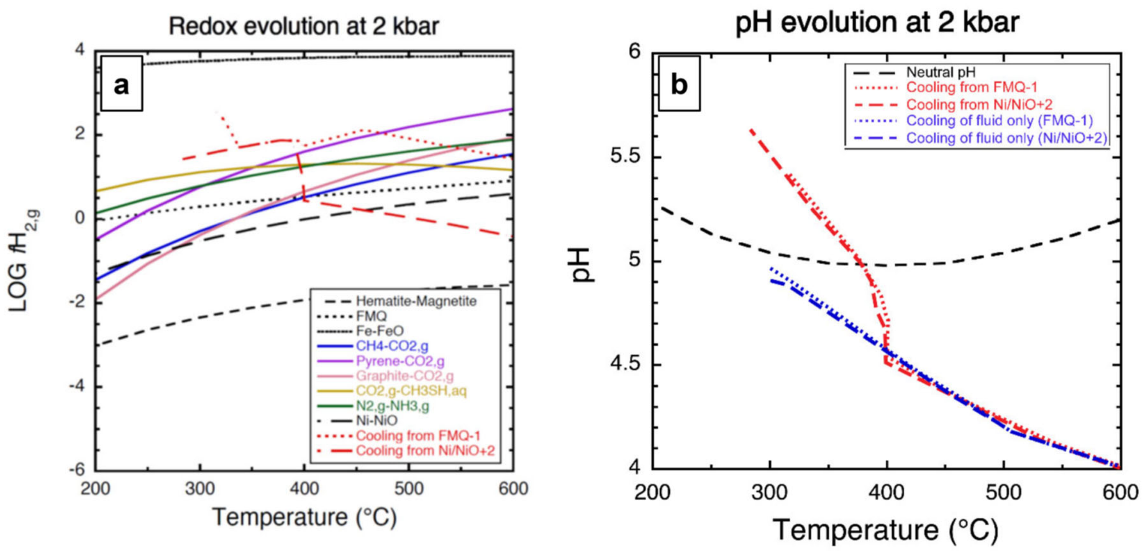

Supplementary Figure 3 | a, Redox evolution, expressed as hydrogen fugacity ($fH_{2,\,g}$), of magmatic fluids starting at log oxygen fugacity ($fO_{2,\,g}$) FMQ-1 or NiNiO+2, during cooling from 600°C to 400°C, and subsequent water-olivine interaction during the main serpentinization stage between 400°C and 300°C. **b,** Associated pH evolution. The fluid evolution without interaction with minerals is also provided for comparison. The fugacities of CO_2 and CH_4 are set to 100, the one of CH_3SH to 10^{-3}, and the ones of N_2 and NH_3 to 1. See Methods for further details. Mineral buffers: FMQ, fayalite-magnetite-quartz-; HM, hematite-magnetite; NiNiO, nickel-nickel oxide.

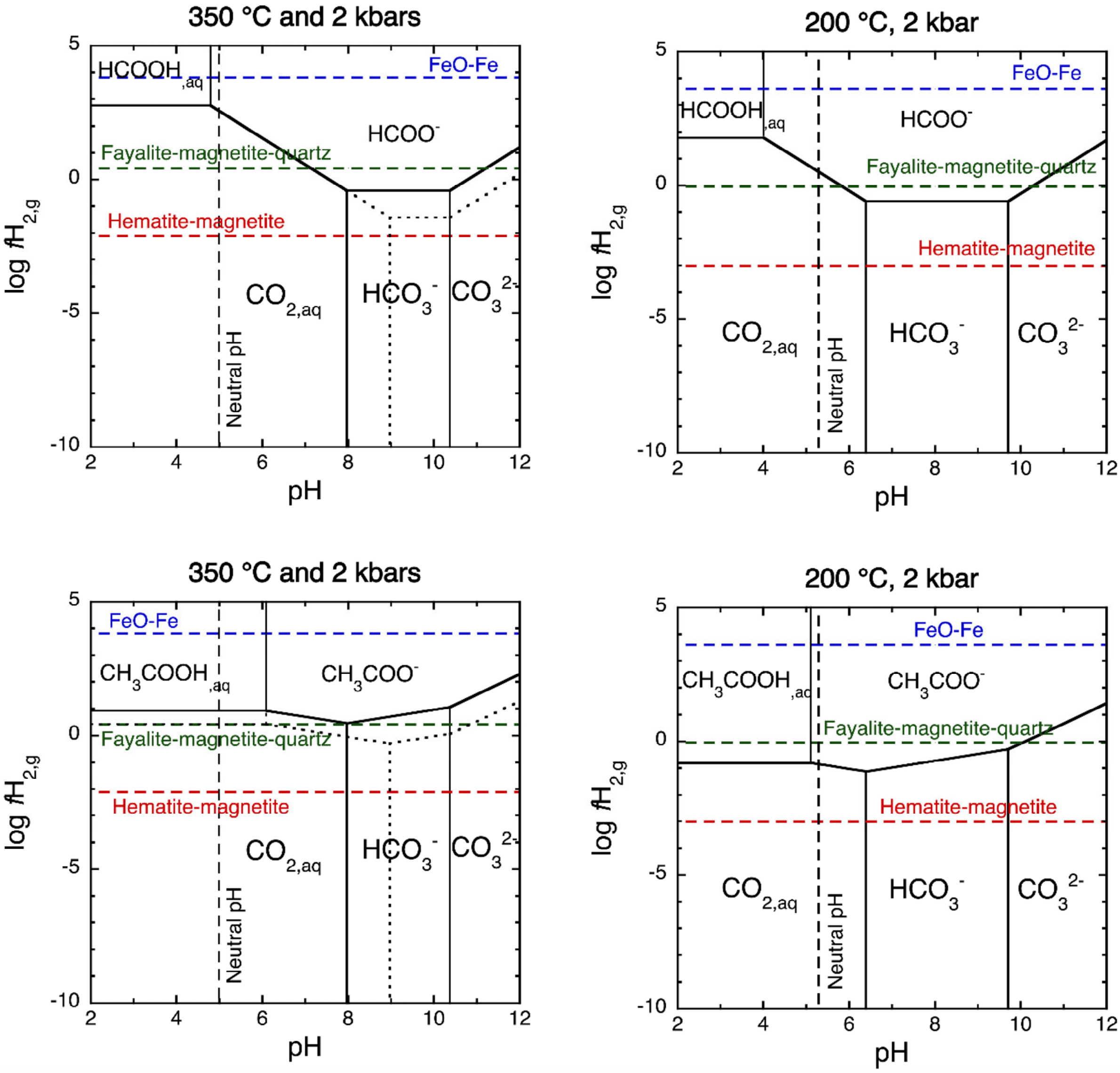

Supplementary Figure 4 | Carbon speciation diagram of the fluid at 200°C and 350°C illustrating the fields of acetic acid and formic acids that extends with decreasing T. During serpentinization, the increase of fH_2 and of pH should shift the fluid toward the fields of organic acids according to the following reactions for formate: $CO_2 + H_2 \leftrightarrows HCOO^- + H^+$, and for acetate: $2CO_2 + 4H_2 \leftrightarrows CH_3COO^- + H^+ + 2H_2O$. The water activity ($aH_2O$) was set to 1 (plain line) or 0.1 (dashed line) and the activities of inorganic and organic carbon were set to 1 and 10^{-2}, respectively.
