# Supplementary material for: The rocky road to organics needs drying
Source: Nat Commun. 2023 Jan 21;14:347. doi: 10.1038/s41467-023-36038-6 (PMC9867705; doi:10.1038/s41467-023-36038-6)
Supplement: Supplementary file 2 — Description of Additional Supplementary Files [file 41467_2023_36038_MOESM2_ESM.pdf]

Description of Additional Supplementary Files:

Supplementary Movie 1: Video of the internal structure of the fluid inclusion FI3 from 3D Raman imaging (see also Fig. 1a and the Methods). The color code is the same as the one of Fig. 3a, but for brucite that is seen in pink here.
